# Technical Note: A comparison of solvents for optimal extraction and morphological identification of blow flies (Diptera: Calliphoridae) from sticky traps

**DOI:** 10.1016/j.fsisyn.2025.100583

**Published:** 2025-04-07

**Authors:** Kate M. Barnes, Mark T. Bulling, Gosia Wosik, Alana F.V. Twinn, Samara Lemon, Chloe Foreman, Kinga Babiarz, Katherine Brown

**Affiliations:** a School of Science, University of Derby, Kedleston Road, DE22 1GB, UK; b School of Criminology and Criminal Justice, University of Portsmouth, Hampshire, PO1 2HY, UK

**Keywords:** Calliphoridae, Trapping, Forensic entomology, Decomposition studies, Taphonomy, Diptera, Insect succession

## Abstract

Passive sampling techniques such as sticky traps are recommended for research studies assessing colonisation patterns of forensically important flies. However, there are no standardised protocols for the optimal removal of flies to ensure accurate morphological identification to species level. This study assessed the use of four freely available solvents (mineral oil, vegetable oil, baby oil and an orange-based solvent) in terms of facilitating extraction from sticky traps, and potential effects on subsequent identification of three blow fly species of forensic importance, *Calliphora vicina, Calliphora vomitoria* and *Lucilia sericata*. Results indicated that species were differentially affected by the oils but, overall, the orange-based solvent had the least effect on the morphological features of each species, and therefore, was considered the best throughout the study. Additionally, the orientation of flies on the traps had no significant effect on the quality of morphological characteristics. It is recommended that the orange-based solvent method outlined in this paper is used for the removal of blow flies from sticky traps.

## 1 Introduction

The determination of the time of death is a vital component of homicide investigations, helping to develop a timeline and to guide police inquiries. Forensic entomology uses the colonisation times of specific insect species to estimate the minimum Post-Mortem Interval (minPMI); the time between the first insect colonisation and discovery of the corpse [1]. Blow flies (Diptera: Calliphoridae) are commonly used in these calculations as they are the primary colonisers of a human corpse in most geographical areas.

Forensic practice is reliant on robust research studies assessing the colonisation patterns of forensically important insects to provide baseline data for calculations of the minPMI. Therefore, use of reliable sampling methods in these studies is essential if accurate colonisation times, insect succession patterns, species diversity and population numbers are to be determined. A variety of passive and active sampling techniques have been used in forensic entomology research. For example, pitfall traps and manual sampling can serve as valuable tools for the comprehensive capture of both beetle and larval fly communities, whereas sticky traps and aerial netting are better suited for capturing adult flies [2,3]. However, there can be inconsistencies between researchers using active techniques due to variation in their experience [3] and in general, the sampling method can significantly affect the number and species of insects sampled [[4], [5], [6]].

Pig carcasses are often used in forensic studies as a good substitute for human corpses [3,7]. The carcasses used can range from stillborn (>1 kg) through to adult size (>60 kg) and, although the insects collected represent the local species composition dynamics throughout decomposition, the smaller sized pigs tend to attract fewer insects than the larger carcasses [[7], [8], [9]]. Additionally, insects on smaller carcasses are also more readily disturbed by active sampling techniques and are less likely to return to the body [5]. Therefore, for forensic entomological studies, it is often more effective to use passive sampling techniques.

Odour-baited traps are a form of passive sampling for flying insects. In particular, considerable research effort has gone into optimising odour-baited traps for blow flies due to their pest status amongst livestock and links with disease [10], as well as their nuisance status in human habitats [11]. Traps baited with varying amounts of animal tissue (70–200g) have proven useful in recording local biodiversity of early colonising flies in forensic studies around the globe [6,[12], [13], [14], [15], [16]]. Whilst useful for establishing local fly community composition, these traps should not be used as a substitute for whole carcasses, as species occurrence and assemblage composition can differ between the two [6,17].

Un-baited traps, such as sticky traps are preferred in decomposition studies so that insect colonisation times can be correlated with specific stages or odours of decay i.e. direct links to the state of the corpse [4,5,18,19]. However, studies that report using sticky traps to collect insects do not always specify the brand of trap, the amount of time it was exposed for or if and how these insects were removed for identification purposes (for example, 19). Reibe and Madea [18] specify using Aeroxon sticky traps with synthetic glue for 30-min periods in their decomposition study using small (1–2 kg) pig carcasses but do not state how the resulting insects were identified. Similarly, Sanford [4] specifies using Catchmaster®, Mouse and Insect Glue Traps for 15-to-60-min periods but does not state if insects were removed from traps for identification purposes. Cruise et al. [5] do not state the brand of sticky traps used but state that they were left *in situ* for 10-min periods four times a day for three days and insects were identified on the traps *in situ*. In the UK, sticky traps often comprise an odourless colourful flower image, coated in a non-specific sticky glue to firmly adhere the flies. They are available cheaply from multiple suppliers and can be left next to the carcass, being quickly collected and replaced at each sampling interval. Due to the high abundance and variety of insects attracted to a carcass, insects often need to be removed from the sticky traps before they can be pinned and reliably morphologically identified to species level using keys such as Sivell [20]. However, the adhesive coating on the traps means that the insects are difficult to remove, and critical morphological characters needed for accurate identification can be obscured, damaged or distorted [21] which is potentially the reason why researchers such as Cruise et al. [5] leave them *in situ* for identification. To date, successful removal of necrophagous adults from these traps has not been reported.

Polar solvents are unsuitable for removing insects from sticky traps [22]. Whilst non-polar solvents (e.g. toluene, heptane and hexane) can be useful for removing sclerotized insects such as Coleoptera and Hymenoptera, they are not appropriate for removing soft-bodied insects such as Lepidoptera [22]. Miller et al. [23] assessed three citrus oils (lemon extract used as a food additive; Histo-clear, an orange based clearing agent; and Livos, a thinning agent) on the removal of several insect taxa. They found them successful for the removal of lepidoptera and other soft bodied insects but the amount and time soaking in the oil depended on the amount of sclerotization of the insect and the condition it was in. Butterwort et al. [21] soaked sticky traps in 600 μl of Histo-clear, whilst shaking for 10–15 min at 60 °C to remove lepidoptera for DNA extraction. This process was repeated before insects were rinsed in 99 % nondenatured ethanol to remove the sticky residue and Histo-clear, air-dried in a laminar flow hood for 15 min and stored in 1 ml of 2 % cetylammoniumbromide (CTAB) solution at −80 °C. Marshall et al. [24] used orange oil to remove glassy-winged sharpshooter leafhoppers (Hemiptera) from yellow sticky traps in Texas, USA. Sticky traps were placed in 2 L of orange oil for 5 min at a time then removed from traps using forceps. These solvents also have the advantages of not leaving the insects brittle and not having any substance toxicity. However, to the best of our knowledge, these oils have not been tested on Calliphoridae, Fanniidae or Muscidae flies, which are frequently key colonisers of dead bodies. Therefore, this study aimed to assess the suitability of four readily available and affordable solvents for effective extraction and morphological identification of three species of forensically important blow flies, *Calliphora vicina* (Robineau-Desvoidy)*, Calliphora vomitoria* (Linnaeus) and *Lucilia sericata* (Meigen). The solvents were chosen based on their availability and affordability in the UK, and non-toxic properties.

## 2 Materials and methods

Two complementary initial pilot studies were conducted at the University of Derby and the University of Portsmouth to assess four solvents (mineral oil, vegetable oil, baby oil and a citrus based solvent) in terms of facilitating extraction from sticky traps, and potential effects on the subsequent identification of the blow fly *Calliphora vicina*.

### 2.1 University of Derby (2022)

**Fig. 1:**
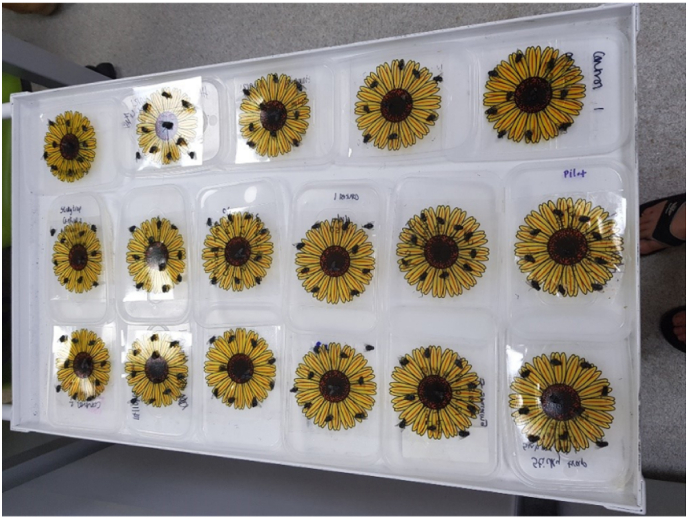
Ten adult *Calliphora vicina* flies were placed onto advanced window fly traps (Rentokil).

From July to September 2022 ten deceased *Calliphora vicina* adult flies were selected at random (mixed sexes) from university laboratory stock populations (<1 month old) and manually placed onto each advanced window fly trap (Rentokil) (Fig. 1). Flies had been deceased for <1 month. Fourteen such traps for each of four oils (orange, mineral, baby and vegetable oil) were made up at least 24 h before each experiment, and every fly was scored using the scoring system shown in Table 1 to assess the morphological quality of the fly. Traps were then immersed in either mineral oil (The Cornish Chopping Board Co.), baby oil (Johnsons), vegetable oil (Morrisons) or a citrus based solvent (Just Orange). Traps were laid flat in a tray (25cmx35cm) containing 250 ml oil to cover the whole trap surface and left immersed for 30 min. If any flies had not separated from the sticky trap within this period, then the trap was left immersed for an additional half an hour. After this point, if the insects were still attached to the sticky traps, they were examined hourly and left overnight if needed. The time taken to remove flies was recorded. Flies were then transferred into 100 ml of 70 % ethanol for 15 min and pinned after removal. Each fly was scored using the scoring system in Table 2. The scores from Table 2 were summed to produce a total score for each fly, which was used in the analysis to test for effects of oil type on the quality of fly following removal.

**Table 1:** Scoring system for blow flies on sticky traps before being submerged in oil for removal. Colour relates to that of abdomen, basicosta and lower calypter, which were scored separately.

Category	Score				
	1	2	3	4	5
**Intactness**	intact	small parts (legs, antenna, wings, bristles) missing	one body part (head/thorax/abdomen) missing	two body parts missing (head/thorax/abdomen)	maximum of one degraded body part remaining
**Colour**	clearly visible	colour dulled but visible	some colour that is sufficient for identification	colour difficult to determine but possible	unable to see colour

**Table 2:** Scoring system for blow flies after being submerged in oil, submerged in ethanol and pinned. Colour relates to that of abdomen, basicosta and lower calypter, which were scored separately.

Category	Score				
	1	2	3	4	5
**Time**	less than 1 h	2 h	3 h	4 h	more than 4 h
**Intactness**	intact	small parts (legs, antenna, wings, bristles) missing	one body part (head/thorax/abdomen) missing	two body parts missing (head/thorax/abdomen)	maximum of one degraded body part remaining
**Colour**	clearly visible	colour dulled but visible	some colour that is sufficient for identification	colour difficult to determine but possible	unable to see colour
**Residue**	no residue	thin layer that does not hide colour or features (bristles)	thin layer that hides some colour but still able to see metallic blue/green	thick layer that hides colour (small patches still visible but not for confident identification of species)	thick layer that hides colour and features fully; colour unable to be seen
**Brittleness**	no brittleness	brittle for small parts (legs, antenna, wings, bristles) to break off	brittle for 1 body part to break off	brittle for 2 body parts to break off	whole body brittle and breaking apart when handled

### 2.2 University of Portsmouth (2022)

From November to December 2022 ten deceased *Calliphora vicina* adult flies were selected at random (mixed sexes) from F1 populations (<1 month old) in Portsmouth and were placed onto one advanced window fly trap (Rentokil) (Fig. 1). Flies had been deceased for <1 month. Nine traps for each of four oil categories (orange, mineral, baby and vegetable oil) were made up at least 24 h before each experiment and each fly on each trap was scored using the scoring system in Table 1. Traps were then immersed in either mineral oil (The Cornish Chopping Board Co.), baby oil (Johnsons), vegetable oil (Morrisons) or a citrus based solvent (Just Orange) by laying traps flat in a tray, and approximately 9 ml of oil was added by a plastic pipette directly on to the flies on the surface for 30 min. If the insects had not separated from the sticky trap within this period, then they were left for an additional half an hour. After this point, if the insects were still attached to the sticky traps, they were examined hourly and left overnight if needed. The time taken to remove flies was recorded. Flies were then transferred to a vial containing 10 ml 70 % ethanol for 15 min, gently inverted in solution, and pinned. After 24 h drying time, each fly was scored using the scoring system in Table 2. The scores from Table 2 were summed for each fly.

In addition to these categories, the work done at the University of Portsmouth also gathered data on the orientation of each fly on the trap (front, back or side), with 10 flies in each orientation, in each oil tested.

### 2.3 University of Derby (2023)

A further study was then conducted to expand the range of blow fly species examined. For this, the pilot study method was repeated using 140 *C. vicina* individuals and applied to 280 *L. sericata* and 280 *C. vomitoria* individuals from May to July 2023.

### 2.4 Data analysis

As data at Derby for *C. vicina* were collected over two years, involving different researchers in the two years, a year (researcher) effect on the scores for individuals of *C. vicina* before being placed on the traps was tested for. A generalized linear model (GLM) with a log link function and Poisson error distribution was applied with the before score as the dependent variable and year and oil as potential additive independent variables. Model assumptions were tested using standard residual diagnostics [24]. Additionally, Cook's distances were used to identify any overly influential data points, and over-dispersion was tested for by comparing residual deviance with a χ^2^ distribution. There was a significant ‘year’ effect (df = 1, LRT = 76.4, p < 0.001). A hierarchical model to account for the ‘year’ effect was not possible as data for only one species was collected in 2022 as part of the pilot study at Derby. Therefore, the *C. vicina* 2022 data were analysed separately from the three species 2023 data from Derby, using the GLM framework described above.

Flies were scored before and after trapping, and therefore there was a lack of independence. As we were interested in the impact of the trapping, the change in score (after – before) was calculated for each fly. 24 (0.86 %) flies in the Derby dataset had a negative value for this difference, and these values were set to zero. For consistency, when analysing the Portsmouth data, only the score categories common to both Portsmouth and Derby were utilised.

In summary, for the 2022 Derby pilot data (*C. vicina* only), the effect of oil on the change in scores was assessed; for the 2023 Derby data (*C. Vicina*, *L. sericata* and *C. vomitoria*), effects of species, oil and species × oil interaction effects were assessed; and for the 2023 Portsmouth data (*C. vicina* only), the additive effects of oil and position were assessed. All analyses used the GLM approach described previously.

## 3 Results

**Fig. 2:**
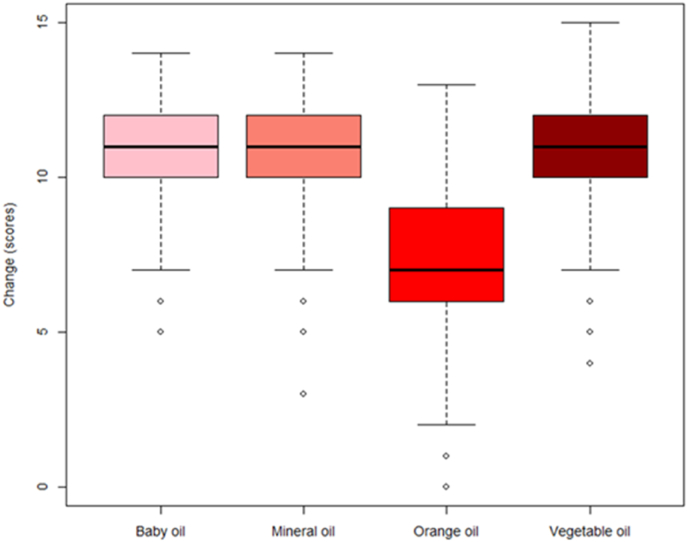
Boxplots showing change (lower values indicate less degradation) in scores by oil categories for data collected at the University of Derby in 2022 *(C. vicina* only).

Analysis of the pilot data from Derby in 2022 indicated that there was a significant effect of oil on the change in scores for *C. vicina* (df = 3, LRT = 153.0, p < 0.001), with orange oil resulting in smaller changes (i.e. less overall degradation) than the for the other oils (p < 0.001 in all comparisons of orange oil with the other oils (baby, vegetable and mineral); Fig. 2).

**Fig. 3:**
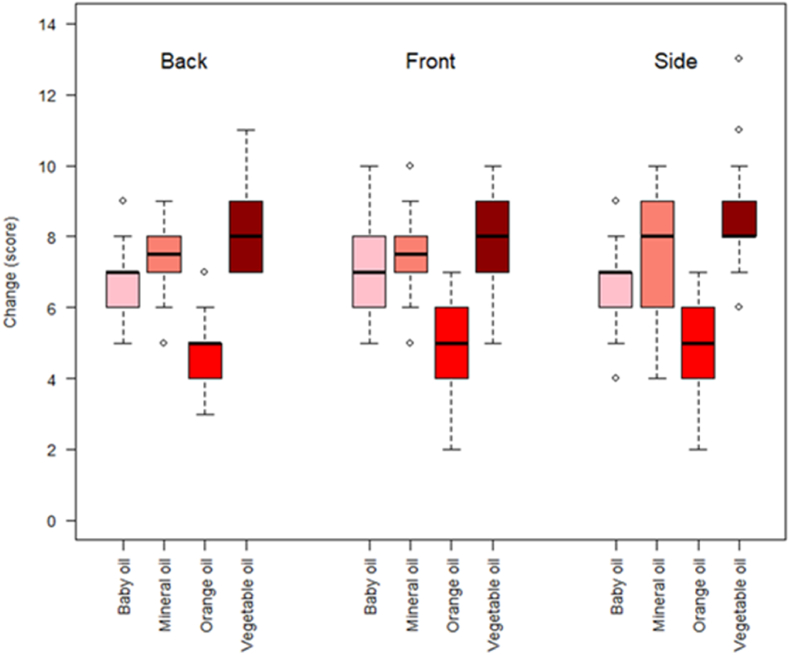
Boxplots showing change (lower values indicate less degradation) in scores within and between oil categories for data collected at the University of Portsmouth in 2022 *(C. vicina* only).

Analysis of data from Portsmouth (*C. vicina* only) indicated no effect of orientation on change in score, but there was an effect of oil (df = 3, LRT = 86.5, p < 0.001). Pairwise comparisons indicated differences in all cases (p < 0.001), with change being lowest with orange oil, followed by baby, mineral and then vegetable oil (Fig. 3).

**Fig. 4:**
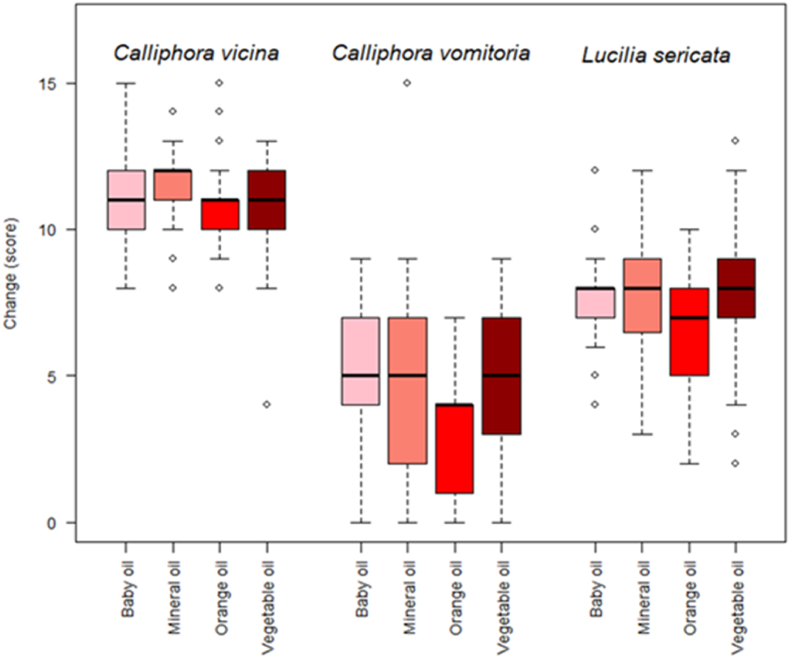
Boxplots showing change (lower values indicate less degradation) in scores within and between oil categories for data collected at the University of Derby in 2023.

The data from the University of Derby in 2023 (all three species) indicated a significant species X oil two-way interaction (df = 6, LRT = 110.3, p < 0.001; Fig. 4). Levels of change (degradation) were lowest for *C. vomitoria* and highest for *C. vicina*, across all the oils. Within species, orange oil tended to result in lower changes in scores, but this was most pronounced in *C. vomitoria* and least pronounced in *C. vicina* (Fig. 4).

**Fig. 5:**
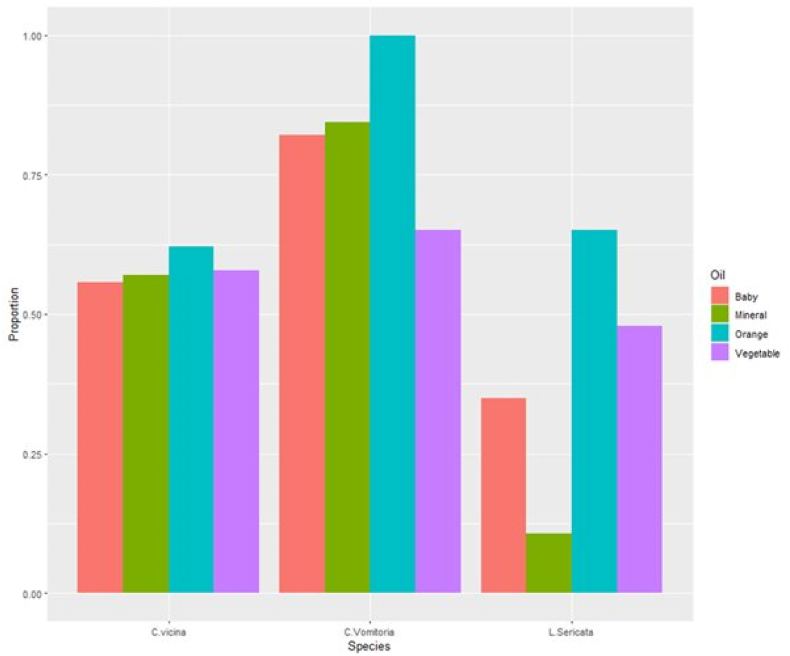
Barcharts of the proportions of flies from the 2023 work at the University of Derby which were considered to be in good enough condition for identification purposes (i.e. scores of 4 or less in all colour ratings for abdomen, basicosta and lower calypter) for each species and oil combination.

As a measure of utility for using the oils to capture flies for identification purposes, we calculated the proportion of flies which had colour scores (Table 2) of less than or equal to four for abdomen, basicosta and lower calypter, after extraction for each of the oils used. A score of four in all of these would be considered sufficient for colour to be good enough for identification purposes. Of the flies utilised in 2023 at the University of Derby, *C. vomitoria* had the highest proportions of useable flies across all oils used, and orange oil consistently gave the highest proportions within all three fly species (Fig. 5). For the flies examined at the University of Portsmouth in 2022 (all *C. vicina*) the lowest proportion was for the baby oil (0.8) with all flies exposed to all the other oils being classified as useable.

## 4 Discussion

This study compared four readily available solvents with respect to the resulting quality of extraction and morphological identification of three species of forensically important blow flies, *Calliphora vicina* (Robineau-Desvoidy)*, Calliphora vomitoria* (Linnaeus) and *Lucilia sericata* (Meigen).

Results across the three studies indicated that the citrus based solvent was the best choice in terms of having the least negative impact overall on the categories tested; time taken to remove the flies from the traps, intactness of specimen, colour of the abdomen, calypter and basicosta, amount of residue left on the specimen and brittleness of specimen (Table 1, Table 2).

In terms of cost involved, the mineral oil was the most expensive at £10.95 a litre, the baby oil was £7 a litre, the citrus-based solvent was £6.40 a litre and the vegetable oil was £1.99 a litre. Therefore, the citrus-based solvent is affordable as well as effective when compared to the other oils in this study. In terms of how this compares to citrus-based solvents used in previous studies, it is more affordable than Histo-Clear ($60/£47.50 per litre) that was used by Butterwort et al. [21] and Miller et al. [23] to successfully remove lepidoptera and other soft bodied insects from sticky traps.

The identification of flies down to species level is fundamental in estimating colonisation times. This study indicated that different species are differentially affected by the oils. For example, *C. vicina* was most negatively affected by the oil process, whilst *C. vomitoria* was least affected. Part of this difference between these two species was due to the different colours of the basicosta [20]. The dark yellow to light brown colour in *C. vicina* had greater potential for change than the black in *C. vomitoria.* However, within *C. vomitoria,* colour degradation in the abdomen and lower calypter did occur, but changes in the lower calypter were much reduced by the orange oil treatment. This highlights that care must be taken when assessing the benefits of different solvents for the extraction of flies from traps as there is the potential for individuals to be generally well preserved, but specific parts may be disproportionately affected, which will be important if these parts are key for identification purposes. Our results indicate that orange oil did not reduce degradation for all components examined, but it never increased it and did reduce it significantly for some components. Therefore, it is recommended as the best of the solvents tested here for extraction purposes.

The current study did not consider the trapping of live flies. Whilst minimal difference is expected given that these data show no effect on morphological characteristics due to orientation, the motion of flies when caught is likely to lead to a more extensive range of orientations than tested here, and result in a) damaged flies prior to removal and b) some more challenging removal. However, the method used in this work with the citrus based solvent was successfully applied to samples from summer 2019, autumn 2022, summer 2023, summer and autumn 2024 after being stored in a freezer following collection from field sites, suggesting that it is likely to be suitable for removal of live caught flies. Identification of these flies to species level using published keys [20,25] has been possible using the citrus-based solvent and included the three species (*C. vicina*, *C. vomitoria* and *L. sericata*) used in this study. Considering that the sticky traps used in this field work were left *in situ* for 24 h periods and flies were still able to be removed and successfully identified to species level indicates that this method could be applied to studies leaving sticky traps out for shorter periods of time (for example, 18, 4–5), where flies might be less entangled in the glue on the trap, and likely less damaged. However, in this study, the effect of the period of time for which the flies were attached to traps and the brand of trap were not assessed and should be investigated in the future.

Blow flies are comparatively physically robust and yet we found that there were substantial differences between the extent of change in quality between species and between solvents used. This suggests that the impact of the solvent will be greater when working with species that are less robust, for example other fly families such as the Fanniidae and Heleomyzidae. This work has clearly shown important consequences for the preservation of specimen quality due to the extraction solvent used, and the work now needs to be extended to other families to identify consistencies and differences in results, providing more specific advice for particular collection scenarios.

## CRediT authorship contribution statement

**Kate M. Barnes:** Writing – review & editing, Writing – original draft, Supervision, Project administration, Methodology, Conceptualization. **Mark T. Bulling:** Writing – review & editing, Software, Methodology, Data curation. **Gosia Wosik:** Investigation. **Alana F.V. Twinn:** Investigation. **Samara Lemon:** Investigation. **Chloe Foreman:** Investigation. **Kinga Babiarz:** Investigation. **Katherine Brown:** Writing – review & editing, Supervision, Project administration, Methodology, Conceptualization.

## Declaration of competing interest

The authors declare that they have no known competing financial interests or personal relationships that could have appeared to influence the work reported in this paper.

## References

[1] Amendt J., Richards C.S., Campobasso C.P., Zehner R., Hall M.J.R. (2011). Forensic entomology: applications and limitations. Forensic Sci. Med. Pathol..

[2] Amendt J., Campobassp C.P., Gaudry E., Reiter C., LeBlanc H.N., Hall M.J.R. (2006). Best practice in forensic entomology – standards and guidelines. Int. J. Leg. Med..

[3] Schoenly K.G., Haskell N.H., Hall R.D., Gbur J.R. (2007). Comparative performance and complementarity of four sampling methods and arthropod preference tests from human and porcine remains at the Forensic Anthropology Center in Knoxville, Tennessee. J. Med. Entomol..

[4] Sanford M.R. (2017). Comparing species composition of passive trapping of adult flies with larval collections from the body during scene-based medicolegal death investigations. Insects.

[5] Cruise A., Hatano E., Watson D.W., Schal C. (2018). Comparison of techniques for sampling adult necrophilous insects from pig carcasses. J. Med. Entomol..

[6] McGonigal H., Jetten K., Brown K. (2022). Annual blowfly attraction and colonisation patterns of liver-baited traps and rabbit carcasses in Southern England. Br. J. Entomol. Nat. Hist..

[7] Tomberlin J. (2012). Assessment of decomposition studies indicates need for standardized and repeatable research methods in forensic entomology. J. Forensic Res..

[8] Kuusela S., Hanski I. (1982). The structure of carrion fly communities: the size and the type of carrion. Ecography.

[9] Hewadikaram K.A., Goff M.L. (1991). Effect of carcass size on rate of decomposition and arthropod succession patterns. Am. J. Forensic Med. Pathol.

[10] Hall M.J.R., Hutchinon R.A., Farkas R., Adams Z.J.O., Wyatt N.P. (2003). A comparison of Lucitraps and sticky targets for sampling the blowfly *Lucilia sericata*. Med. Vet. Entomol..

[11] Dadour I.R., Cook D.F. (1992). The effectiveness of four commercial fly traps at catching insects. J. Aust. Entomol. Soc..

[12] Hwang C., Turner B.D. (2005). Spatial and temporal variability of necrophagous Diptera from urban to rural areas. Med. Vet. Entomol..

[13] Brundage A., Bros S., Honda J.Y. (2011). Seasonal and habitat abundance and distribution of some forensically important blow flies (Diptera: Calliphoridae) in Central California. Forensic Sci. Int..

[14] George K.A., Archer M.S., Toop T. (2013). Abiotic environmental factors influencing blowfly colonisation patterns in the field. Forensic Sci. Int..

[15] Farinha A., Dourado C.G., Centeio N., Oliveira A.R., Dias D., Rebelo M.T. (2014). Small bait traps as accurate predictors of dipteran early colonizers in forensic studies. J. Insect Sci..

[16] Barnes K.M., Grace K., Bulling M.T. (2015). Nocturnal oviposition behavior of forensically important Diptera in central england. J. Forensic Sci..

[17] LeBlanc K., Boudreau D.R., Moreau G. (2021). Small bait traps may not accurately reflect the composition of necrophagous Diptera associated to remains. Insects.

[18] Reibe S., Madea B. (2010). How promptly do blowflies colonise fresh carcasses? A study comparing indoor with outdoor locations. Forensic Sci. Int..

[19] Albushabaa A.H.H., Almousawy H.R. (2016). Insect succession and carcass decomposition during spring and summer in An-Najaf province-Iraq. Res. J. Pharmaceut. Biol. Chem. Sci..

[20] Sivell O. (2021).

[21] Butterwort V., Dansby H., Zink F.A., Tembrock L.R., Giligan T.M., Godoy A., Braswell W.E., Kawahara A.Y. (2022). A DNA extraction method for insects from sticky traps: targeting a low abundance pest, phthorimaea absoluta (Lepidoptera: gelechiidae), in mixed species communities. Mol. Entomol..

[22] Murphy W.L. (1985).

[23] Miller R.S., Passoa S., Waltz R.D., Mastro V. (1993). Insect removal from sticky traps using a citrus oil solvent. Entomol. News.

[24] Zuur A.F., Leno E.N., Smith G.M. (2007).

[25] Szpila K., Gennard D. (2012). Forensic Entomology-An Introduction.

